# Updates on the Effect of Mycotoxins on Male Reproductive Efficiency in Mammals

**DOI:** 10.3390/toxins11090515

**Published:** 2019-09-03

**Authors:** Diala El. Khoury, Salma Fayjaloun, Marc Nassar, Joseph Sahakian, Pauline Y. Aad

**Affiliations:** Department of Sciences, Faculty of Natural and Applied Sciences, Notre Dame University-Louaize, Zouk Mosbeh 2207, Lebanon

**Keywords:** mycotoxins, spermatogenesis, spermiogenomics, infertility, epigenetics

## Abstract

Mycotoxins are ubiquitous and unavoidable harmful fungal products with the ability to cause disease in both animals and humans, and are found in almost all types of foods, with a greater prevalence in hot humid environments. These mycotoxins vary greatly in structure and biochemical effects; therefore, by better understanding the toxicological and pathological aspects of mycotoxins, we can be better equipped to fight the diseases, as well as the biological and economic devastations, they induce. Multiple studies point to the association between a recent increase in male infertility and the increased occurrence of these mycotoxins in the environment. Furthermore, understanding how mycotoxins may induce an accumulation of epimutations during parental lifetimes can shed light on their implications with respect to fertility and reproductive efficiency. By acknowledging the diversity of mycotoxin molecular function and mode of action, this review aims to address the current limited knowledge on the effects of these chemicals on spermatogenesis and the various endocrine and epigenetics patterns associated with their disruptions.

## 1. Underestimated Potent Environmental Disruptor: Mycotoxins

Mycotoxins are a heterogeneous group of low molecular weight toxic fungal products with the ability to cause disease in humans and other vertebrates [1,2,3,4]. They are ubiquitous and unavoidable harmful agents [5] found in almost all types of foods, including cereals [6] and wheat derivatives [7], animal [8] and dairy [9] products, fruits [10], and even wine [11,12]. Owing to the vast differences in their structure and biochemical effects [1], some mycotoxins are more prevalent in certain countries and continents than others. In fact, African countries tend to have higher levels of mycotoxin contamination [13,14] due to the optimal conditions for fungal growth such as high temperatures, elevated moisture levels, and lack of proper hygienic measures [13]. A 2012 study reports that 96% of Tunisian and 50% of Moroccan staple foods were contaminated with mycotoxins [15], most commonly by nivalenol and beauvericin. Similarly alarming, a large percentage of processed feeds in Asia and the Americas, feeds in Europe, wheat from Australia [16], and edibles in Lebanon [17] all tested positive for mycotoxins. Furthermore, the average dietary exposure levels to ochratoxin A (OTA) and deoxynivalenol (DON) in a Lebanese urban population far exceeded the toxicological reference values (TRVs) [18]. By better understanding the toxicological and pathological aspects of mycotoxins, we can be better equipped to fight these diseases, and the biological and economic devastation they might induce.

Mycotoxins pose a major health hazard to both animals and humans in general, and a recent increase in male infertility has been associated with the increased occurrence of these mycotoxins in the environment [19]. Infertility rates have worsened from 42 to 48.5 million couples worldwide between 1990 and 2010 [20], affecting 1 out of 7 couples trying to conceive [21]. In fact, a 1% yearly average decline of sperm concentration was recorded in semen quality studies between 1938 and 1991 [20,22,23], alongside decreased sperm parameters and total motile sperm count (TMSC), and increased morphological abnormalities [22,23]. This alarmingly continuous decline of sperm count and human fertility worldwide are attributed to many factors, among which, the significant role of endocrine-disrupting chemicals (EDCs), such as mycotoxins and pesticides [24,25], are not well established.

Although an international scientific consensus on sperm count rate was not achieved throughout the 1970s, a comprehensive review [20] covering a 50-year longitudinal study reported irrefutable evidence of declining semen quality. As several studies attempted to tackle the potential causes of this decline, Bahadur et al. [26] suggested environmental pollution and lifestyle factors as decisive influences on reproductive health with a possible endocrine underlying cause, a possible intimation of epigenetics. Since then, an accumulating body of evidence suggests that pre-conceptional exposure to lifestyle and environmental factors impact the phenotype of the current and subsequent generations through epigenetic mechanisms and developmental plasticity [27]. Dietary habits [28], starvation [29], psychological traumas [30], alcohol consumption [31], smoking [32], toxins [33], physical activity [34], and other emerging factors have all been implicated in influencing the phenotype of organisms and their progeny. These non-genetic interventions, specifically though regulatory mechanisms known as epigenetics, regulate gene expression rather than induce gene mutations [35]. Evidence is leaning toward these mechanisms in compromising the phenotype of the next generation through the remodeling of the epigenetic blueprint of spermatozoa [36]. Cells respond to environmental stressors through increased epigenetic responsiveness and variations providing an adaptive capacity for living things and a developmental plasticity for their future progeny [37]. Further, under the influence of parental or congenital environments, chromatin dynamics can fluctuate between permissive and repressive states to control gene expression transiently or heritably [38]. The sources capable of inducing such epigenetic deregulation can be traced back to internal factors, such as inflammation [39], the microbiome [40], and aging [41], and less widely studied lifestyle and external exposures such as cannabis [42]; pollution and chemical agents [43]; nutrition [44]; fungal toxins, such as Aflatoxins [45,46,47,48,49,50]; ochratoxin [51,52,53] and DON [54]; and endocrine disruptors [55]. In fact, aberrant epigenetic modifications could be the source of many serious human diseases, syndromes, and developmental complications [56]; dysregulated methylation of promoters may silence critical genes involved in tumor suppression, as in the case of hepatocellular carcinoma [57], breast cancer [58], and leukemia [59], or it may disrupt important signaling pathways leading to psychiatric and mental disorders, such as schizophrenia [60] and autism [61], or cardiovascular and metabolic diseases, and even contribute to male infertility [62]. Although the causes of male infertility still remain elusive despite ongoing and extensive investigations, recent studies associate it to epigenetic abnormalities in chromatin states [63], sperm-borne miRNAs [64], and methylation levels of *PIWIL1/2* alleles [65], and insinuate possible transgenerational implications [66]. Therefore, both genetic and epigenetic toxicology studies can help in revealing the causes behind decreasing semen quality, and possibly establish the effect of mycotoxins on reproductive health. With the diversity of mycotoxin molecular function and mode of action, this review aims at addressing the current limited knowledge on the effects of these chemicals on spermatogenesis and the various endocrine patterns associated with their disruptions.

## 2. Spermatogenesis: A Complex and Delicate Process

In mammals, spermatogenesis is a complex process involving the division and differentiation of spermatogonial stem cells into mature spermatozoa [67,68,69,70,71] that takes place in the convoluted seminiferous tubules of the testis [70,71]. The seminiferous tubules are pipe-like structures containing both germ and supporting somatic cells [72,73], which are surrounded by testosterone-producing Leydig cells and blood vessels [74]. Leydig cells are responsible for the production of testosterone [75], without which, spermatogenesis would not advance beyond meiosis [76], while Sertoli cells control the environmental milieu of tubules and facilitate differentiation of spermatozoa via direct contact [77]. With the proper support and signaling from the Sertoli and Leydig cells, germ cells undergo a stepwise differentiation and maturation process from the basement membrane of the seminiferous tubules to the lumen where the differentiated germ cells [72,73] are released into the rete testes as spermatozoa, then travel through the epididymis in preparation for capacitation and ejaculation as mature sperm cells [68,74,77,78]. This process occurs in three major phases, namely spermatocytogenesis, meiosis, and spermiogenesis [68,72]. During spermatocytogenesis, germ cells undergo a cycle of numerous mitotic divisions, generating a cell population from which some contribute to the renewal and maintenance of the stem cell population, while others differentiate to produce spermatogonia and primary spermatocytes [67,68,69] in the meiosis phase. This stage is marked with the duplication and exchange of genetic material crucial for genetic diversity; two successive cell divisions reduce the chromosome number in half and yield four haploid round spermatids [67,68,69]. Subsequently, the last phase of spermiogenesis induces the differentiation of these round spermatids into completely mature, though non-fertilizing, spermatozoa [67,68,69]. The intricate nature of this entire process makes it prone to several types of disruptions at multiple time points, which can jeopardize the quantity and/or quality of spermatogenic yield, leading to reproductive complications [79].

The hypothalamo–pituitary–gonadal (HPG) axis acts in concert through feedback loops to orchestrate the crucial events of spermatogenesis. Gonadotropin releasing hormone (GnRH), a hormone periodically released by the hypothalamus, stimulates the pituitary to release Luteinizing (LH) and Follicle Stimulation (FSH) hormones [80] in a pulsatile fashion [81]. LH stimulates the Leydig cells to produce testosterone [80], which subsequently acts as a negative feedback loop on GnRH production [82]. Altogether, these hormones control steroidogenesis, the complex, multi-enzymatic process through which cholesterol is converted into biologically active steroid hormones [83] such as testosterone secreted by the testicular cells [84]. This process is mediated by the steroidogenic Sertoli and Leydig cells, which express the cholesterol side-chain cleavage enzyme (P450scc) [85]. Steroidogenesis starts with the conversion of cholesterol to pregnenolone by P450scc [83], a slow-acting, rate-limiting enzyme [86], followed by its conversion to progesterone by 3β-Hydroxysteroid dehydrogenase (3βHSD) or to 17α-hydroxypregnenolone by P450c17 [87,88,89]. 3βHSD is also involved in the conversion of 17α-hydroxypregnenolone to 17α-hydroxyprogesterone (17OHP), dehydroepiandrosterone (DHEA) to androstenedione, and androstenediol to testosterone [90]. Other reactions include the conversion of testosterone to dihydrotestosterone by 5α-reductases or to estrogens by aromatases (P450aro) [91,92].

Highlighting the multi-enzymatic and hormonal aspects of spermatogenesis is important to understand the environmental disturbances affecting male infertility, where mycotoxins have been shown to interfere at various levels and disrupt the activity of P450scc, 3βHSDs, 5α-reductases, and/or P450aro in both males and females.

### 2.1. Effect of Mycotoxins on Fertility

With a clearly complex yet delicate balance of enzymatic and hormonal control of steroidogenesis, it is important to understand the minute impact of mycotoxins on such processes. Unfortunately, research on the effect of mycotoxin in males, specifically on spermatogenesis [93,94,95], steroidogenesis [96], or even the HPG axis [97,98,99,100], is scarcely available in the literature. Few clues drawn from the effect of various mycotoxins on steroidogenesis from female studies [101,102,103,104] portray the biological threat mycotoxins can engender [105]. DON can reduce epididymal, seminal vesicle, and prostate weights; spermatid count; and serum testosterone concentrations, while inducing sperm tail and nuclear morphology abnormalities, in a dose dependent fashion in rats [106,107]. In females, DON inhibited estradiol and progesterone secretion in bovine granulosa cells, increased oocyte and granulosa cell apoptosis, reduced porcine oocyte maturation capacity via autophagy, and induced aberrant epigenetic modifications [96,108]. Furthermore, zearalenone (ZEA) has been found to induce a dose-dependent reduction of aromatase, P450scc, and 3βHSD transcripts in cultured porcine granulosa cells from porcine ovaries [104,109], while beauvericin inhibited estradiol and progesterone synthesis in bovine granulosa cells [101], and showed antagonism toward progesterone cell lines, where it decreased the binding of progesterone to its receptor [110]. In addition, beauvericin exerted potent cytotoxic effects on lung cell surrogates [111] and ovarian hamster cells [112]. Furthermore, in vitro cultured porcine oocytes and embryos exposed to physiological levels of beauvericin showed damaged development [113]. Altogether, the few studies on mycotoxin’s effect on the female reproductive systems establish mycotoxins as possible endocrine disruptors; similar negative reproductive effects in the male reproductive system are anticipated, as reviewed in the following sections.

### 2.2. Effect of Mycotoxins on Sertoli Cells

The research linking mycotoxins specifically to Sertoli cells is scarce and mostly involves the mycotoxins as summarized in Table 1. Furthermore, the diversity of models and dosage used does not allow for a clear determination of the specific mycotoxin effect on Sertoli cell physiology, gene expression, and dynamics.

For example, zearalanone (ZEA) is an estrogenic mycotoxin found to damage Sertoli cells and potentially induce apoptosis [117,118,119] in both mice and rat. The induction of apoptosis and necrosis of rat Sertoli cells by ZEA via extrinsic and intrinsic apoptotic pathways suggest that its reproductive toxicity may be navigated by multiple pathways [119]. The addition of the anti-estrogen ICI 182.780 [117] or other antioxidant enzymes [124] inhibited the effects of ZEA in adult rats [117] and mice [124] neonatally exposed to ZEA, suggesting that its effects are at least partially modulated by its estrogenic activity. Moreover, ZEA treatment can damage the cytoskeletal structure and affect the specific secretory functions of Sertoli cells, which may be an underlying cause of ZEA-induced reproductive toxicity [118].

### 2.3. Effect of Mycotoxins on Leydig Cells

Mycotoxins also act directly on Leydig cells, disrupting crucial enzymatic and hormonal activities. Table 2 shows the potent steroidogenic and cytotoxic effects of various mycotoxins on Leydig cells. Specifically, aflatoxin B1 (AFB1) can directly reduce testosterone concentration in a dose-dependent manner and inhibit the expression of 3βHSD and 17β-hydroxysteroid dehydrogenase enzymes (HSD17B3) [125]. In mice, AFB1 upregulated renin mRNA along with 193 extracellular matrix and signaling genes, 49 signal transduction genes, 45 immune regulation genes, and 230 cell differentiation genes in the testis [126]. Thus, AFB1 exhibits wide-ranging effects on mRNA expression, but whether this translates into anything meaningful in terms of protein expression remains to be elucidated. Citrinin (CTN) also reduced testosterone levels [127] by inducing the apoptosis of Leydig cells, possibly via p53 expression and activation of multiple caspases. Furthermore, T-2 decreased testosterone levels in mice in a dose-dependent manner [128]. Similarly, ZEA caused a dose- and time-dependent inhibition of testosterone stimulated both by suppression of hCG (10 ng/mL) [128,129] and cAMP [130], while DON exhibited the most cytotoxicity out of seven other tested mycotoxins [131] in MA-10 murine Leydig cell lines.

### 2.4. Effect of Mycotoxins on Spermatogenesis

Spermatogonial stem cells (SSCs) are at the foundation of spermatogenesis and male fertility. Similar to other tissue-specific stem cell niches, SSCs are rare, representing only 0.03% of all germ cells in rodent testes [135]. Thus, any cytotoxic effect of mycotoxins on this small percentage of cells can compromise all consecutive processes. An exhaustive search of the literature on the effect of various mycotoxins on SSCs are summarized in Table 3, and show a potent dose and time dependent effect of mycotoxins on these SSCs.

The nonsteroidal estrogenic mycotoxin ZEA is known to cause toxicity within the testes of male rats [136]. Histopathology of ZEA-treated mice (5 mg/kg BW (body weight)) revealed that 12 h after treatment, germ cell degeneration occurred in stages I–VI with the damaged germ cells, especially spermatogonia and spermatocytes, gradually undergoing apoptosis. Also, daily DON exposure for 28 days via gastric intubation of male rats reduced body weight, feed consumption, and epididymal and seminal vesicle weights, while increasing germ cell degeneration, sperm retention, and abnormal nuclear morphology [106]. Similar detrimental effects were seen in adult male mice exposed to a daily intraperitoneal injection of CTN for 7 days with an increase in the number of abnormal spermatozoa and a decrease in the number of live spermatozoa in a dose-dependent manner [137].

Furthermore, intramuscular injection of increasing doses of AFB1 (10, 20, or 50 µg/kg BW) in adult rats resulted in the reduction of reproductive organ weights, daily sperm production, epididymal sperm count, viable sperm, and motile sperm [138]. Similarly, the T-2 toxin showed an increase in the number of abnormal spermatozoa and a decrease in spermatozoa count in adult male mice, and these resulted in low pregnancy rates and high fetal resorption after exposure to 10 and 15 mg/kg BW T-2 toxin [144].

## 3. Epigenetic Implications

As previously discussed, mycotoxins are implicated in negatively affecting male and female reproductive physiology, altering fertility. Furthermore, epigenetics, the recent field of multigenerational outer-chromosomal inheritance, is gaining momentum in the literature. Therefore, introducing epigenetic mechanisms and the effect of environmental contaminants is imperative to setting the stage for our reviewing the available literature on the epigenetic impact of mycotoxins on male infertility, spermatogenesis, and steroidogenesis. Very few studies have addressed the effect of mycotoxins directly on the male reproductive epigenome. Altogether, epigenetic implications in any process, especially a lagging field of mycotoxin effects on male infertility, is essential to gaining a full scope on the topic and further consideration during future research planning.

### 3.1. Epigenetic Mechanisms and Environmental Exposure

**Figure 1:**
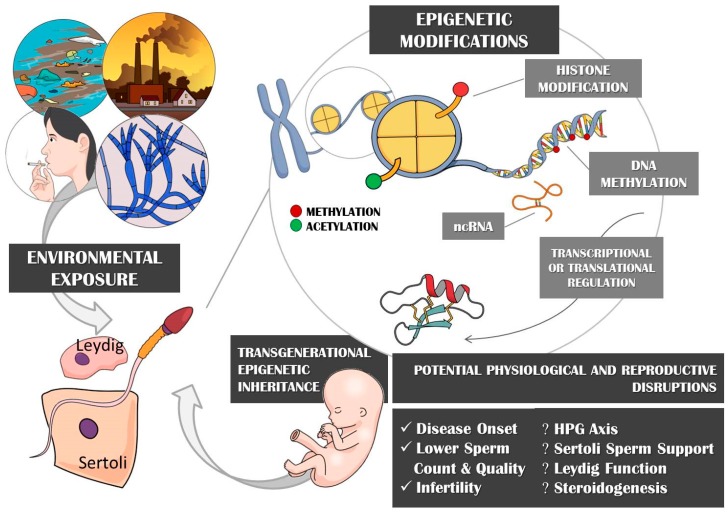
Epigenetic involvement in transgenerational reproductive control.

Epigenetics comprise changes in phenotype resulting from differential gene regulation rather than DNA sequence alterations, with these epigenetic regulations playing critical and ubiquitous roles in a wide range of cellular and developmental processes [146]. Recent advances in this field relate epigenetic mechanisms to covalent modifications of DNA (cytosine methylation and hydroxymethylation) [147] and histones (lysine acetylation, lysine and arginine methylation, serine and threonine phosphorylation, and lysine ubiquitination and sumoylation) [148], production of RNA transcripts (miRNA, mRNA, and sRNA) [149], prions [150], and nucleosome positioning [151]. These different control mechanisms, as summarized in Figure 1, can simultaneously be recruited in response to environmental stressors.

The most widely studied of the epigenetic modifications is DNA methylation; it plays a critical role in important cellular and developmental processes such as cell differentiation and embryonic development [152]. DNA methyltransferases (DNMTs) are the main components involved in methylating DNA, though other enzymes have also been discovered [153]. The main targets of methylation are the cytosines of CpG islands found near promoters, which can modulate the expression of a given gene, although non-CpG methylation has been observed in embryonic stem cells and neural development [154]. Histone modifications have also been implicated in diverse biological processes such as gene regulation, DNA repair, chromosome condensation (mitosis), and spermatogenesis (meiosis) [155]. Despite the identification of several histone modifications, the functional understanding of these modifications remains unclear. In addition to their control of gene expression, miRNAs received a recent highlight due to their involvement in altering the stress reactivity of the zygote post-fertilization. When sires were exposed to chronic paternal stress, they showed sperm-borne miRNAs alterations with a mechanistic role [156]. In addition to the different regulatory mechanisms, epigenetic patterns can also be altered directly on the DNA by bioactive compounds or indirectly by affecting the enzymes that catalyze DNA methylation and histone modification [157,158], which would explain the diversity of epigenetic responses. Nutrients and bioactive substances, such as retinoic acid, resveratrol, curcumin, sulforphane, and tea polyphenols [159], have been postulated to adopt both strategies. Adding a further complexity to this process suggests that these environmental effects can induce specific or unspecific DNA methylation changes that might affect genetic pathways directly or indirectly. Hence, the multiple mechanisms adopted by epigenetic control can have a global and devastating effect on the organism, which explains the fact that aberrant epigenetic modifications have been correlated with diverse human diseases, syndromes, and developmental defects.

### 3.2. Contribution of Epigenetics in Environmentally-Induced Disease Predisposition

Although the study of epigenetics initially focused on the role it plays as a regulatory system, recent landmark observations have linked environmental exposure and gene–environment interactions to a significant proportion of human malignancies. The Dutch Hunger Winter remains a classic example of such environmental interactions where pregnant women during the famine period gave birth to children and future generations who exhibited higher rates of obesity, diabetes, schizophrenia, and mortality upon adulthood [144]. The latter set forward the study of transgenerational epigenetic inheritance, where the germline undergoes epigenetic modifications inherited by future generations despite the extensive epigenetic resetting event, with epigenetic tags on some loci associated with metabolic and neurological disorders that can escape this reprogramming [160].

### 3.3. Epigenetics Involvement in Germline Modulation and Infertility

Investigations of paternal effects demonstrated further that environmental factors are capable of inducing changes in the sperm, and this in turn would affect the organism itself and modulate the developmental programming of the offspring by transgenerational epigenetic inheritance. A high-fat diet given to rats induced modified epigenetic sperm profiles; metabolic dysfunction was apparent throughout two generations [161]. Embryonic male rats exposed to the endocrine disruptor vinclozolin in utero (through maternal administration) manifested adult-onset diseases in the first generation that persisted in four subsequent generations; sperm epigenome modification following treatment at the time of gonadal sex determination enabled this transgenerational transmission. Furthermore, changes in DNA methylation of the male germline resulted in transcriptional changes in several tissues, such as testes, brain, and prostate, which consequently led to adult-onset pathologies including testicular, prostate, and renal abnormalities, and increased incidence of tumors [162,163,164]. The persistence of these epigenetic signatures is contingent with the type and timing of epimutations that could intervene throughout several windows of vulnerability [165,166] and generate different types of epigenetic responses. Furthermore, an array-based DNA methylation profiling in male infertility established the role of allele-specific DNA hypermethylation of *PIWIL1/2* involved in RNA-mediated gene silencing [55]. Sperm of mice brought up on a folate-deficient diet showed altered methylation patterns for genes associated with chronic diseases, autism, and development [137]. Moreover, sperm quality and pregnancy rate were improved when males were supplemented with vitamins, such as methyl-group donor folic acid and micronutrients, that might be involved in modifying the epigenome [138]. While alcohol consumption induces a global and unspecific methylation profile change [139], male rats sustained on a high-fructose diet showed modified DNA methylation at peroxisome proliferator-activated receptor *α* (*PPARα*) and carnitine palmitoyltransferase 1Acarnitine palmitoyltransferase 1A (*CPT1A*) promoter regions in their liver [140] and male mice on a low-protein diet had offspring showing modified key lipid and cholesterol biosynthesis genes in the liver [141], which may have resulted from specific modified DNA methylation of the paternal germline.

### 3.4. Epigenetic Effects of Mycotoxins in Disease and Infertility

The effect of mycotoxins on the modulations of the epigenome as they relate to the male reproductive system and infertility are not well studied. Depending on the timing of the epimutations (germline or zygote), tissues originating from these modified cells would harbor alterations predisposing or leading to a wide range of malignancies [167,168]. For example, long-term exposure to low doses of the carcinogenic AFB1 induces persistent epigenetic changes in primary human hepatocytes, promoting the development of hepatocellular carcinoma [159]. On the other hand, fumonisin B1 exclusively caused DNA hypermethylation in C6 glioma cells associated with human esophageal cancer [143]. Recently, exposure of pregnant women to the mycotoxin Zearalenone/Zearalenol was associated with disease susceptibilities of the progeny [129]. Furthermore, in utero exposure to some mycotoxins severely compromised postnatal development of neonatal rats and delayed testes descent, and it impaired both steroidogenesis and spermatogenesis, inducing a suppressed reproduction at adulthood [160,161]. Additionally, a mycotoxin-induced miRNA modification in various tissues targeted specific genes and increased DNA damage, proliferation, apoptosis, homeostasis, cancer, migration, oxidative stress, and detoxification [132]. Further, OTA revealed deregulated DNA methylation, ncRNA production, and histone modifications associated with cell apoptosis, oxidative stress, cell autophagy, and protein synthesis inhibition [133], whereas histone acetyltransferases regulated OTA toxicity and carcinogenicity [131].

Altogether, these limited studies indicate the possible involvement of mycotoxins in altering the various female and male reproductive cells, be it directly acting on the germline or indirectly via a steroid-like function. However, the targeted role of a given mycotoxin on the various components in male spermatogenesis requires further investigation.

### 3.5. Transgenerational Epigenetic Inheritance through Imprinted Genes

Both internal and external factors are capable of disturbing the crucial genetic and epigenetic regulation involved in germline development such as the complex process of spermatogenesis. Germline epimutations are capable of inducing drastic implications on an organism compared to epigenetic mosaicism where modifications are contained within specific types of tissues rather than expressed in the whole body [169,170]. During the intricate process of spermatogenesis, germ cells undergo crucial chromatin and step-wise epigenetic changes marked with differential DNA methylation patterns, global shifts in histone post-translational modifications, and production of certain miRNAs that together induce their gradual differentiation into functional sperm cells [171]. The mammalian germline undergoes an extensive remodeling and epigenetic reprogramming that is paramount for imprinting and regulating embryogenesis [172]. Two major reprogramming events underline mammalian development: primordial germ cells (PGCs) go through an almost genome-wide methylation erasure that is hypothesized to wipe out all cellular memory to reach totipotency [173], following which, the fertilized zygote is re-methylated de novo throughout its gradual cleavage and differentiation [174]. Nonetheless, this initial methylation erasure is incomplete to allow some intact and crucial genomic features (escapees) to evade this event [175]; functional analysis of these regions reveals several critical genes involved in brain and neuronal development [176]. However, this event also opens a dangerous developmental time window susceptible to the transfer of environmentally altered epigenetic marks or insults that may impact fertility and embryonic competence [177]. Cannabis (THC) exposure seems to alter methylation patterns of genes in the sperm of rats and humans, causing lower sperm concentrations with a possible transgenerational aftermath [42]. Further, cannabis alters sperm count [178], and even modifies the sperm itself [42], with a significant overlap between THC target genes in rat sperm and aberrantly methylated genes in the brain of rats born to parents exposed to THC during adolescence [42]. During each round of spermatogenesis (SSC to sperm), epigenetic patterns and chromatin states are re-established to generate efficient mature sperm [179]; however, this process is prone to errors and epimutations induced from either internal or environmental sources [180].

A chronic unhealthy lifestyle (lack of exercise, smoking, and drinking) can induce epimutational accumulation in SSCs and contribute to decreased male fertility and poor transgenerational outcomes [21,181,182]. SSCs undergoing clonal amplification before entering meiosis and differentiation can accumulate aberrant epigenetic modifications, which can be carried on to the mature sperm, increasing the chances of these epimutations to be transferred to the next generation [183]. Associations between chemotherapy exposure and aberrant changes in sperm parameters and epigenetic mutations in the spermatogonial stem cell population may compromise human sperm integrity and potentially be transmitted to future generations [184]. Even in vitro processes, such as embryonic stem cell differentiation into SSCs, have been shown to epigenetically alter the germline and promote abnormalities transgenerationally in mice [185,186], further raising a serious question regarding the long-term safety or efficiency of therapeutic stem-cell-based applications. One type of epimutation can transpire through DNA methylation, which in normal circumstances is responsible for important physiological roles such as X inactivation and genomic imprinting [187]. Inheritance and expression of traits associated with imprinted genes is regulated through epigenetic marks; imprinting causes only one copy of the genes to be functional while the other one is silenced in a parent-of-origin manner [188,189]. This monoallelical expression compromised by mutations or epimutations poses more serious implications than biallelically expressed genes; aberrant sperm DNA methylation of imprinted genes is linked to spermatogenic impairments and abnormalities [190,191,192].

Many imprinted genes are clustered and regulated by the single imprinting control region (ICR) [190]. ICRs are part of regulatory regions known as differentially methylated regions (DMRs) that entail discrete DNA elements with a heritable spot useful for distinguishing parental origin [193]. Epigenetic aberrations in imprinted genes have been associated with adverse effects on cancer [194], embryogenesis, nervous system development, and DNA repair [172,195]. Subfertile males harbored dysregulated sperm methylation profiles associated with abnormal sperm parameters [196]; specifically, hypomethylation of H19 and hypermethylation of SNRPN imprint control regions [196,197,198,199], which is exacerbated further by cigarette smoking [200]. Therefore, exposure to reproductive environmental toxins during critical windows of mammalian development can trigger irreversible and heritable epigenetic tags.

Male mice treated with low and high concentrations of ZEA show altered expressions of testicular genes involved in methylation such as *Ccnd1*, *Kdm4a*, and *Spata2* [95]. Similar effects were seen in human subjects exposed to Bisphenol A (BPA) where sperm DNA hydroxymethylation of several known sperm functional and sperm associated genes were involved in embryonic stem cell differentiation, growth, and early development [201].

## 4. Challenges to the Study of the Effect of Mycotoxins on Male Spermatogenesis

Challenges to studying the effect of mycotoxins on the male reproductive cells and spermatogenesis are multifaceted, ranging from the established toxicity of these substances to their vast diversity and effects, and to the lack of an easy isolation, purification, and culture system for spermatogenic cells.

First, the toxicity levels of many mycotoxins have not been established in many of the model species, especially farm animals where the majority of the effects are expected as these mycotoxin infections are becoming ubiquitous in farm operations. Many studies [117,124,134,202,203], though not reviewed herein, have focused on antagonizing the effect of these mycotoxins, and many products are marketed for farm animal application to counter the mycotoxin effects. Added to this scarcity of investigations, the majority of the studies observed these mycotoxins as disruptors in females [100,101,102,103,104], and only a few in males [93,94,95,143,204,205], with most of these studies observing the effect of mycotoxins in animal models rather than in cell culture.

Second, isolating Sertoli or Leydig cells is generally a time-consuming and very delicate process that also necessitates the availability of fresh tissue, complex enzymatic digestion steps of the seminiferous tubules, followed by segregation of the various cells using flow cytometry and adhesion purification techniques such as DSA-lectin or gradient centrifugation. Moreover, with spermatogonial stem cells (SSCs) culture being essential to male infertility therapy in humans [206], endangered species preservation, and transgenic animal technology, isolation of these cells have been exhaustively described in the literature [206,207,208,209,210,211,212,213,214], though the survival of these cells beyond 48 to 96 hours in culture is challenging [211,212,215] as they require continuous hormonal or co-culture stimulation. Additionally, identifying the various cells before culture can prove challenging [207,208,210,212,213,216,217], thus many adopt marker-assisted selection following culture, though this technique only reports a percentage of each cell type in the culture.

Therefore, benchmarking of the isolation, purification, and culture of male reproductive cells is essential to determining the effect of individual mycotoxins on spermatogenesis, steroidogenesis, toxicity, and epigenetic marks; this benchmarking will help better decipher the long-term impact farm animals and humans are experiencing when exposed to mycotoxins.

## 5. Conclusion and Future Directions

Spermatogenesis is a complex process involving a multitude of cells and an interdialogue between neuroendocrine processes. Therefore, any minor disruption of any of the players might lead to dire consequences, including alteration of sperm quality and quantity, infertility, or inconspicuous modifications of the genome or epigenome. With many mycotoxins presenting steroidogenic-like and toxic effects, further focus on male spermatogenesis and the various players and cells involved is needed in order to better understand the immediate reproductive consequences, as well as the risks to future generations. Understanding how epimutations accumulate during parental lifetimes can shed light on their implications regarding fertility, reproductive competence and outcome, and offspring health. Due to the nature of spermatogenesis marked by continuous cycles of mitosis and meiosis, adult males are more prone to accumulating and storing environmentally induced epigenetic alterations than females. Although recent technological advances for epigenetic profiling have been significant, there still remains a need for a systematic understanding of how epigenetics shapes cellular circuitry and disease pathogenesis. These epigenetic modifications may provide possible molecular justifications to understand heritability or predisposing factors observed in some diseases. With the limited amount of research on the effects of mycotoxins and EDCs on male reproduction and fertility, and the challenges associated with culturing the various cells involved, better isolation and culture techniques and further studies are required to determine the crucial time of exposure to environmental toxins and identify factors that result in germline-transmitted adult-onset diseases and those that have an epigenetic basis.

## Figures and Tables

**Table 1:** Effects of mycotoxins on Sertoli cells.

Mycotoxin	Species	Dose	Exposure	Main Findings with Respect to Sertoli Cells	Ref.
CTN	Mouse	0–200 μM	6–72 h	Decreased cell viability and proliferation Increased apoptosis, and necrosis in a dose-dependent manner	[114]
DON	Mice	10 ppm	90 days	No effect on relative testis weight and testicular spermatid counts No effect on the number of Sertoli cells in the seminiferous tubules	[107]
FB1	Rabbit	0.13–10 mg/kg diet *	196 days	Degeneration of Sertoli cell	[115]
OTA	Mice TM4	0–5 μM	24 h	Decreased proliferation Dose-dependent phosphorylation of PI3K (Akt, P70S6K, and S6) and MAPK (ERK1/2 and JNK) pathways	[94]
T-2	SerW3 cells	0.012–1.2 μg/mL (0.025–25.72 μM)	24–48 h	Increased cytotoxicity in a dose-dependent manner Targets blood-testis barrier in vitro	[116]
ZEA	Rat	0–10 nM	48 h	Negatively influenced spermatogenesis and male fertility ZEA effect inhibited by in vitro addition of anti-estrogen (ICI 182.780) → ZEA estrogenic activity	[117]
		0–20 g/mL	24 h	Damages the cytoskeletal structure Disrupts specific secretory functions	[118]
		0–200 μM	6–36 h	Induces apoptosis and necrosis via extrinsic and intrinsic apoptotic pathways	[119]
		0–20 μmol/L (0–62.3 μM)		Induces apoptosis Activates the Fas-Fas ligand signaling pathway Regulates mitochondrial apoptosis pathway	[120]
		20 mg/kg BW *	5 weeks	Increased serum prolactin No effect on testis weights, serum luteinizing hormone, and follicle-stimulating hormone	[121]
		4 or 40 μg	16 days	Weak estrogen effect on Sertoli cell development in pre-pubertal rats	[122]
	Mice TM4	0–100 μM	24 h	TM4 cell cycle G2/M arrest Apoptosis through ROS- and ER-stress and the ATP/AMPK pathway	[123]

* In vivo studies fed a set amount per Kg of Body weight; CTN—citrinin; DON—deoxynivalenol; FB1—fumonisin B1, OTA—ochratoxin A; T-2 = trichothecene-2; ZEA—zearalenone. In parentheses, measures converted to μM.

**Table 2:** Effects of mycotoxins on Leydig cells.

Mycotoxin	Species	Dose	Exposure	Main Findings with Respect to Leydig Cells	Ref.
AFB1	Mouse	50 μg/kg BW *	45 days	Upregulation of genes involved in cell differentiation, extracellular space, and immunity	[126]
	Rat	0–10 μM	35 days	Extra-hepatic toxicity by inhibition of proteins involved in androgen biosynthesis such as StAR, HSDB3, and HSD17B3	[125]
CTN		50 and 100 μM	36 h	Reduced testosterone secretion Induced apoptosis	[127]
T-2	Mouse	1–10^2^ μM	24 h	Dose-dependent decrease in testosterone levels	[128]
ZEA		0–20 μg/mL (0–62.3 μM)	1–24 h	Dose- and time-dependent inhibition of testosterone stimulated by both hCG and cAMP	[130]
		0.01–100 μM	24 h	Suppressed hCG-induced testosterone secretion	[129]
		5 μM	24 h	Modified mitochondrial lipid metabolism Increased energy production Inhibited steroidogenesis and esterification	[132]
		0–200 μg/mL (0–623 μM)	24 h	ER stress pathway activated in ZEA-induced apoptosis	[133]
	Rat	2.5–20 μg/mL (7.8–62.3 μM)	12 h	Investigation of anti-ZEA compounds	[134]

* In vivo study; AFB1—aflatoxin B1; CTN—citrinin; T-2—trichothecene-2; ZEA—zearalenone. In parentheses, measures converted to μM.

**Table 3:** Effect of mycotoxins on spermatogenesis in vivo.

Toxin	Species	Exposure	Daily Dose *	Effect on Spermatogenesis	Ref.
AFB1	Rats	60 days	10–50 µg	Reduction of reproductive organ weights and sperm quantity and quality Decreased steroidogenesis	[138]
		48 days	0.8–3.2 ppm	Dose-dependent decrease of developing spermatozoa in seminiferous tubules	[139]
CTN	Mice	7 days	0.0625–6.25 mg	Increased abnormal spermatozoa Decreased live spermatozoa number and count, and serum testosterone	[137]
DON	Rats	28 days	0.5–5 mg	Decreased testicular spermatid numbers Increased germ cell degeneration, sperm retention, and abnormal nuclear morphology	[106]
FB1	Pigs	6 months	0.2–15 mg	Reduced testicular and epididymal sperm reserves Reduced daily sperm production No influence on the relative weights and volume of the testes or epididymis	[140,141]
	Rabbits	175 days	0.13–10 mg	Delayed puberty, impaired semen quality and spermatogenesis, and induced embryo mortality	[142]
OTA	Rats	8 weeks	289 µg	Decrease in stages I and VII germ cells Increase in stages XII and XIII germ cells	[93]
Patulin	Rats	60–90 days	0.1 mg	Increased sperm counts Decreased sperm counts	[143]
T-2	Mice	7 days	0–15 mg	Increased abnormal spermatozoa Decreased testicular and cauda epididymal sperm counts, efficiency of sperm production, and serum testosterone concentrations	[144]
ZEA	Rats	48 h	5 mg	Germ cell degeneration, especially spermatogonia and spermatocytes	[145]
	Mice	7 days	0–75 mg	Dose-dependent reduction of testicular and cauda epididymal sperm counts and serum testosterone	[129]

AFB1—aflatoxin B1; CTN—citrinin; DON—deoxynivalenol; FB1—fumonisin B1, OTA—ochratoxin A; T-2—trichothecene-2; ZEA—zearalenone; * Per kg body weight, except ppm in water per animal.
